# CREB activity in dopamine D1 receptor expressing neurons regulates cocaine-induced behavioral effects

**DOI:** 10.3389/fnbeh.2014.00212

**Published:** 2014-06-11

**Authors:** Ainhoa Bilbao, Claus Rieker, Nazzareno Cannella, Rosanna Parlato, Slawomir Golda, Marcin Piechota, Michal Korostynski, David Engblom, Ryszard Przewlocki, Günther Schütz, Rainer Spanagel, Jan R. Parkitna

**Affiliations:** ^1^Institute of Psychopharmacology, Central Institute of Mental Health, Faculty of Medicine Mannheim, University of HeidelbergHeidelberg, Germany; ^2^Department of Molecular Biology of the Cell I, DKFZ-ZMBH Alliance, German Cancer Research CenterHeidelberg, Germany; ^3^Institute of Applied Physiology, University of UlmUlm, Germany; ^4^Department of Medical Biology, Institute of Anatomy and Cell Biology, University of HeidelbergHeidelberg, Germany; ^5^Department of Molecular Neuropharmacology, Institute of Pharmacology of the Polish Academy of SciencesKrakow, Poland

**Keywords:** CREB, dominant negative CREB, dopamine receptor D1, activity-dependent gene expression, cocaine-related behavior, addiction

## Abstract

It is suggested that striatal cAMP responsive element binding protein (CREB) regulates sensitivity to psychostimulants. To test the cell-specificity of this hypothesis we examined the effects of a dominant-negative CREB protein variant expressed in dopamine receptor D1 (D1R) neurons on cocaine-induced behaviors. A transgenic mouse strain was generated by pronuclear injection of a BAC-derived transgene harboring the A-CREB sequence under the control of the D1R gene promoter. Compared to wild-type, drug-naïve mutants showed moderate alterations in gene expression, especially a reduction in basal levels of activity-regulated transcripts such as *Arc* and *Egr2*. The behavioral responses to cocaine were elevated in mutant mice. Locomotor activity after acute treatment, psychomotor sensitization after intermittent drug injections and the conditioned locomotion after saline treatment were increased compared to wild-type littermates. Transgenic mice had significantly higher cocaine conditioned place preference, displayed normal extinction of the conditioned preference, but showed an augmented cocaine-seeking response following priming-induced reinstatement. This enhanced cocaine-seeking response was associated with increased levels of activity-regulated transcripts and prodynorphin. The primary reinforcing effects of cocaine were not altered in the mutant mice as they did not differ from wild-type in cocaine self-administration under a fixed ratio schedule at the training dose. Collectively, our data indicate that expression of a dominant-negative CREB variant exclusively in neurons expressing D1R is sufficient to recapitulate the previously reported behavioral phenotypes associated with virally expressed dominant-negative CREB.

## Introduction

Development of addictive behavior and drug reward-seeking processes involve the learning and the formation of long-lasting conditioned associations. These learning processes are associated with drug-induced synaptic plasticity and cellular adaptations within the brain reward system, in particular the medium spiny neurons (MSNs) of the nucleus accumbens (NAc) (Hyman et al., 2006; Russo et al., 2010; Lüscher and Malenka, 2011; Pascoli et al., 2011; Stuber et al., 2011).

The transcription factor cAMP responsive element binding protein (CREB) is regarded as a key mediator of drug-induced adaptations which are of relevance for the development of addictive behavior (Robison and Nestler, 2011). This has been demonstrated by the direct manipulation of CREB activity within the nucleus accumbens (NAc) and its impact on cocaine-induced responses. Thus the expression of dominant negative mutant forms of CREB in the NAc and dorsal striatum results in alterations in the motivational and psychomotor properties of cocaine (Carlezon et al., 1998; Barrot et al., 2002; Fasano et al., 2009). Particularly, it was shown that dominant negative CREB enhances sensitivity to cocaine and conditioned place preference (CPP) at low doses (Carlezon et al., 1998) without having an effect on cocaine self-administration (Larson et al., 2011). Transgenic mice with an overexpression of dominant negative CREB in basal forebrain were also more sensitive to the rewarding effects of intracranial self-stimulation (Dinieri et al., 2009). On the other hand, viral-induced elevations of CREB within the NAc shell of the rat induced an aversion to environmental cues that were previously paired with low doses of cocaine and in less sensitivity to the rewarding effects of the drug at higher doses (Carlezon et al., 1998). The same viral vector treatment increased cocaine self-administration (Larson et al., 2011).

However, the extent at which CREB transcription mechanism is responsible for the described cocaine-mediated behavioral responses is controversial. Thus, while genetically inhibiting CREB function increases cocaine-induced responses those manipulations may not necessarily be sufficient to abolish activity-dependent transcription in the striatum or hippocampus (Blendy et al., 1995; Lemberger et al., 2008). We have previously proposed that discrepancies between CREB-mediated transcriptional activation and behavioral consequences in response to drugs of abuse may be due to the difference in the methodology used to block CREB activity which either fails to block CREB activity completely or may induce compensation by cAMP responsive element modulator (CREM) (Bilbao et al., 2008). Alternatively, virus-based approaches used in previous studies inactivated all neurons in the injection area, and it therefore remains unclear which neuronal population—in the reward pathway- is responsible for CREB-dependent drug effects.

In this study we wanted to address these methodological problems by generating a novel transgenic mouse line in which the A-CREB protein—a dominant-negative protein with very high affinity to the CREB family of proteins (Ahn et al., 1998; Jancic et al., 2009)—is expressed under the control of the dopamine receptor D1 (D1R) gene promoter (D1-A-CREB strain). As shown here introduction of this transgene does not induce compensation by CREM and provides neuronal specificity to D1R expressing neurons. Utilizing these mice our findings show differential roles for CREB in D1R expressing neurons in the behavioral versus transcriptional responses induced by cocaine.

## Materials and methods

### Mouse generation

We generated transgenic mice expressing a dominant negative CREB protein (A-CREB) under control of the mouse D1R gene (*Drd1*a) following the previously described procedure (Parkitna et al., 2009). In short, the construct was recombined into a bacterial artificial chromosome (BAC; RP24–179E13) harboring the mouse *Drd1a* gene. The BAC was purified, the vector sequences were removed, and the transgene was injected into the pronuclei of fertilized oocytes from C57BL/6N mice. A-CREB contains the leucine zipper of CREB plus an acidic extension that enhances the affinity for, and disrupts the DNA-binding activity of, CREB family members (CREB, CREM, ATF-1) but no other bZIP proteins 12). Experimental animals were generated by crossing D1-A-CREB transgenic mice (+/T) to C57BL/6N mice (+/+). Transgenic animals were genotyped using the following primers: agg gca ttt gga gag atg tg and tct gac ttg tgg cag taa agg. Southern blot analysis was used to determine the number of transgene copies integrated in a single locus, as described previously (Parlato et al., 2006). Transgenic mice were maintained as congenic with the C57BL/6N strain.

### Immunohistochemistry and *in situ* hybridization

For immunohistochemistry and *in situ* hybridization, dissected brains were fixed for 48 h in 4% paraformaldehyde and then cut with a vibratome (Leica, Wetzlar, Germany) at 50 μm. Free-floating sections were processed for *in situ* hybridization as described previously (Parkitna et al., 2010). The following antibodies were used: tyrosine hydroxylase (TH) (1:2000; Millipore Corporation, Billerica, MA, USA), D1R (1:3000; Sigma-Aldrich Corp., St. Louis, MO, USA), NeuN (1:3000; Millipore Corporation, Billerica, MA, USA), cleaved caspase-3 (1:1000; Cell Signaling Technology Inc., Danvers, MA, USA), Dynorphin (1:1000; Neuromics, Edina, MA, USA), FLAG (1:1000; Sigma-Aldrich Corp., St. Louis, MO, USA).

### Expression profiling

Array gene expression profiling was performed using the MouseWG-6 v2 BeadChip arrays (Illumina Inc., San Diego, CA, USA) according to the manufacturer's instructions and following the procedure described previously (Piechota et al., 2010). RNA samples were prepared from the striata of 5 naïve D1-A-CREB mice and 5 control animals as follows: brains were fixed overnight in RNAlater at 4°C, then sliced on a vibratome (Leica, Wetzlar, Germany) at 150 μm and the striatum, including the NAc, was microdissected with needles under a binocular. Total RNA was prepared by the method of (Chomczynski and Sacchi, 2006) and its quality was assessed on RNA LabChips (Agilent Technologies, Santa Clara, CA, USA). Microarray quality control was performed using the BeadArray R package from the Bioconductor suite (Gentleman et al., 2004). After background subtraction, the data were normalized using quantile normalization and then log2-transformed. Statistical analysis of most significant differences was performed with the gene set enrichment analysis (GSEA) suite using the signal-to-noise metric (Subramanian et al., 2005).

Measurements of selected activity-dependent transcripts were performed in D1-A-CREB and wild-type mice after the reinstatement of CPP 1 h after injection of 7.5 mg/kg cocaine. RNA isolation was performed following the same procedure as in the case of array gene expression analysis. RNA was reverse-transcribed with a modified MMLV (Omniscript, Qiagen) and then used for real-time PCR with fluorescent probes for target detection (TaqMan, Applied Biosystems, Foster City, CA, USA). The abundance of *Arc*, *Fos*, *Fosb*, *Egr1*, *Egr2*, *Per1*, *Npas4*, *Pdyn*, and *Crem* was measured. Additionally as house-keeping control *Hprt* was used to verify sample uniformity.

### Behavioral procedures

#### Animals

Male wild-type and D1-A-CREB mice (minimum 8 weeks old) were maintained on a 12–12 h light-dark cycle (with lights on at 7:00 AM) under controlled temperature (21 ± 2°C) and humidity (50 ± 5%) conditions. For all studies, mice were single housed and received *ad libitum* access to food and water. Experiments were conducted in accordance with European Union guidelines on the care and use of laboratory animals, and were approved by the local animal care committee (Karlsruhe, Germany).

#### Measurement of locomotor activity, anxiety- and depression-like behavior

***Homecage activity.*** Diurnal locomotor activity in the home cage was monitored by using an infrared sensor (Mouse-E-Motion; Infra-E-Motion GmbH, Henstedt-Ulzburg, Germany). A Mouse-E-Motion device was placed above each cage (30 cm from the bottom), so that the mouse could be detected at any position inside the cage. The device was sampling every 4 s whether the mouse moved or not. The sensor could detect body movements of the mouse of ≥1.5 cm from one sample point to the next. Monitoring of locomotor activity started before the beginning of the experiments and lasted for 3–4 days, and data were collected every 4 h to measure the diurnal pattern of locomotor activity.

***Habituation to the activity box.*** This test was used to assess animal exploratory activity and reactivity to novel environment and to evaluate the effects of habituation mechanisms. Animals were placed in activity chambers in which locomotor activity was measured each minute for a period of 30 min. Clear Plexiglas boxes (40 × 40 × 40 cm) were used, and the locomotor activity was measured with a TruScan activity monitoring system (Coulbourn Instruments, Allentown, PA, USA).

***Elevated plus maze.*** The plus maze consisted of 2 open arms and 2 closed arms extending from a central platform. The maze was elevated 50 cm above the floor and illuminated from the top at 60 lux. Each mouse was placed at the intersection of the 4 arms of the maze and allowed to explore all 4 arms freely for 5 min, and the behavior was recorded and measured by the Noldus/EthoVision 3.1 monitoring system (Wageningen, The Netherlands).

***Light-dark box.*** The light-dark box test consisted of black and white compartment (45 × 20 × 27 cm). The dark compartment (15 × 20 × 27 cm) was covered and the light compartment (30 × 20 × 27 cm) remained open, and was kept at a luminosity of 350 lux. A door was located in the wall between the two chambers allowing free access between the light and dark compartments. Each mouse was placed in the dark chamber and was allowed to explore the box for 5 min, and the behavior was recorded and measured by the Noldus/EthoVision 3.1 monitoring system (Wageningen, The Netherlands).

***Sucrose preference.*** Mice were divided into two groups to test the preference for a 1 or 5% sucrose concentration solution, respectively. Prior to the test, mice were habituated to the corresponding solution by replacing the water bottle by a bottle containing the sucrose solution for 30 min in the homecage. Two days later, preference for sucrose vs. water was tested in a two-bottle free choice test for 15 min. Preference was calculated as percent sucrose solution intake to total liquid intake.

***Forced Swimming Test (FST).*** This test was performed as previously described (Dong et al., 2011). In a pre-test session, mice were forced to swim individually for 6 min in a glass beaker (basin height: 10 cm, 21°C). Twenty four hours later the mice were retested in identical conditions. The FST data presented were collected during the second, retest session. Both FST sessions were videotaped. The mouse was considered immobile when it floats motionlessly or made only those movements necessary to keep its head above the water surface. The total duration of the immobility during the last 4 min of the 6 min test was recorded.

#### Cocaine-induced behaviors

***Dose-response effect on locomotor activity.*** Cocaine hydrochloride (Sigma-Aldrich Chemie GmbH, Munich, Germany) was dissolved in saline and administered i.p. at doses of 0, 5, 10, and 20 mg/kg. Immediately following injection mice were exposed to the activity chambers where locomotor activity was measured during 30 min.

***Conditioned place preference (CPP), extinction and reinstatement of cocaine-seeking behavior.*** The procedure of acquisition, extinction and reinstatement of cocaine-induced CPP was adapted from our original description (Engblom et al., 2008). The CPP paradigm consisted of three different phases: preconditioning, conditioning and drug-free test. For the preconditioning, the mice were injected with saline and immediately placed in the conditioning boxes for 20 min and allowed to explore the apparatus. During conditioning phase, mice were treated during 8 days with alternating injections of cocaine (5 or 10 mg/kg, i.p.) or saline, and confined into the corresponding compartment immediately after the injection for 30 min. For the expression or drug-free test, the mice were allowed to explore the whole apparatus without any treatment on day 9. Once CPP was established, mice underwent extinction. Once the conditioning was extinguished, the mice were given a priming injection of cocaine (3 and 7.5 mg/kg, respectively, i.p.) and had free access to the entire compartment for 20 min (on day 19).

***Development and expression of behavioral sensitization and conditioned locomotion.*** As in our previous studies (e.g., Engblom et al., 2008), during the CPP procedure, the effects of repeated cocaine injections on locomotion were assessed by comparing the distance traveled during the first drug-paired and the first non-drug-paired trials, and the expression of behavioral sensitization was assessed during the priming-induced reinstatement test. The conditioned locomotion was also measured during the first drug-free CPP expression test, which was restricted to the cocaine-paired compartment and excluded the locomotion displayed in the saline and central compartments.

***Cocaine self-administration (CSA).*** Behavioral training and testing were performed in mouse conditioning chambers (Med Associates; model ENV-307W) as described previously (Novak et al., 2010). Chambers were placed inside sound and light attenuating cubicles. Each chamber was equipped with two retractable levers, a cue light above each lever, and a house light located on the opposite wall. During cocaine self-administration, a polyethylene/PVC tube connected the implanted catheter, via a swivel (Instech Solomon), to an infusion pump (PHM-100, Med Associates) located outside of the cubicle. Reinforcement consisted in 36 μl of cocaine solution (0.5 mg/kg/infusion) delivered along 4 s. For the cocaine self-administration (CSA) baseline training, mice underwent CSA for 6 days/week, without any previous lever training for food pellets. CSA sessions were 2 h long, and started with the presentation of the two levers and the house light being turned on. A press on the active lever under a fixed ratio 1 (FR1) schedule of responses was reinforced with a drug infusion paired with the illumination of the cue-light above the lever for the entire duration of the infusion (4 s). Reinforcement was followed by a time-out period (16 s) during which further active lever responses were recorded but had no consequence. The house light was turned off at the beginning of the infusion and remained off for the whole infusion and time-out period (20 s in total). Inactive lever responses were recorded throughout the session, but had no scheduled consequence.

### Statistics for behavioral experiments

All the statistical treatment of the data was conducted using one- or Two-Way ANOVA, with a repeated measures factor when necessary, followed by Newman-Keul's *post-hoc* tests, when appropriate.

## Results

### Generation and characterization of D1-A-CREB transgenic mice

**Figure 1:**
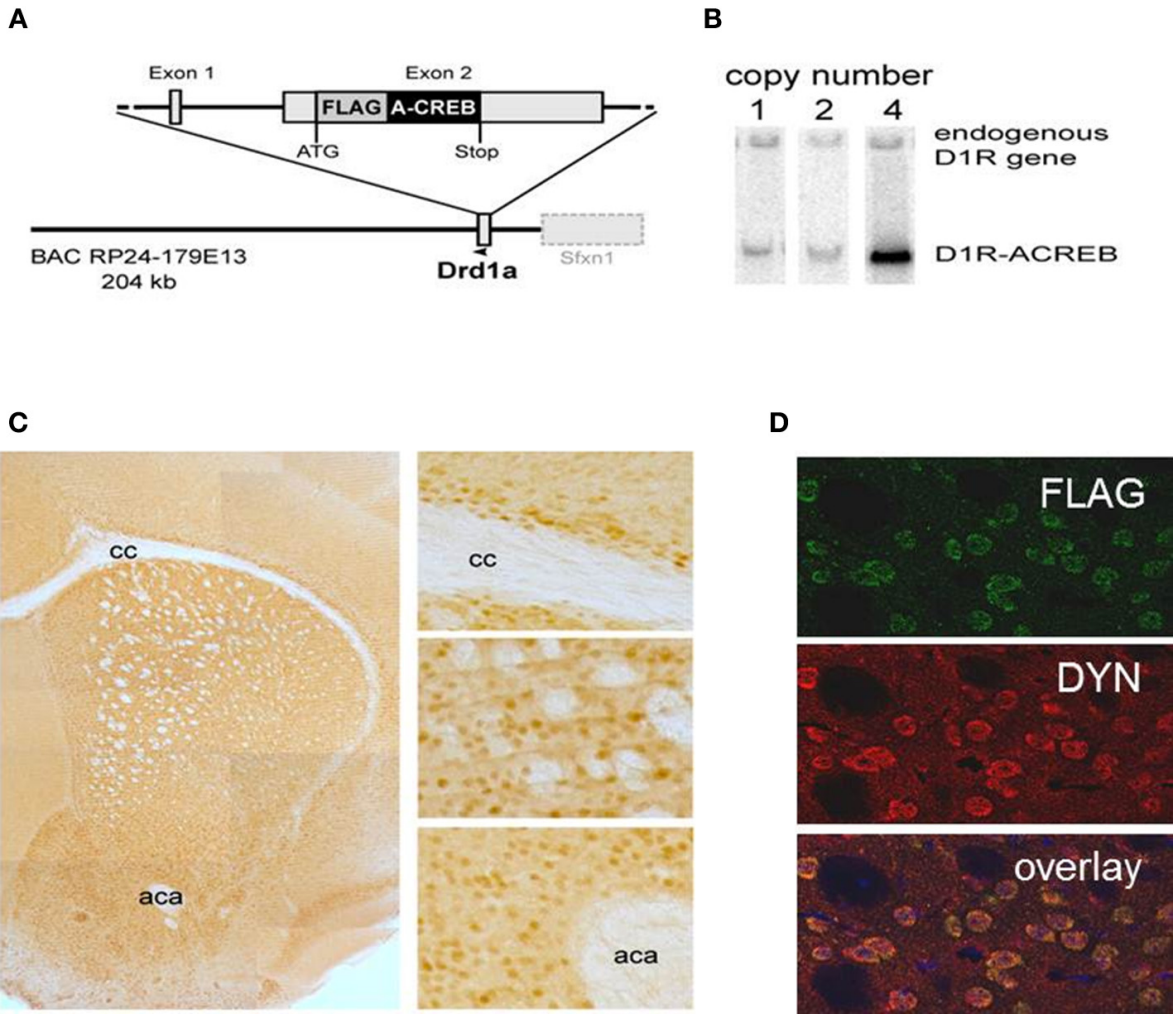
**Cell-type specificity of the A-CREB transgene. (A)** Design of the transgene D1R-A-CREB BAC construct. This construct was inserted after the translational start of the gene encoding the dopamine D1 receptor in a BAC. **(B)** Southern blot of 3 founder lines of D1R-A-CREB with 1, 2, and 4 copies of the transgene. **(C)** Expression of the transgene in D1R-A-CREB mice in coronal brain sections. Brain slides were incubated with anti-Flag antibodies and a peroxidase-conjugated secondary antibody and stained with 3,3′-diaminobenzidine (overview). The D1-A-CREB construct was expressed in layer VI of the cortex (upper), striatum (middle) and NAc (lower). **(D)** Specific expression of A-CREB in dynorphin-expressing neurons as shown by double-immunoflorescence using anti-FLAG (green) and anti-dynorphin (red) show perfect overlay. Scale bars: **(C)** 30 μm; **(D)** 15 μm. cc, corpus callosum; aca, anterior commissure.

To test the role of CREB in D1R expressing neurons we generated transgenic mice with a selective inactivation of CREB in these neurons (D1-A-CREB mice). The sequence encoding the A-CREB (Ahn et al., 1998) was cloned into a BAC construct containing the D1R gene (*Drd1a*; Figure 1A) using the recombineering procedure (Liu et al., 2003), as described before (Parkitna et al., 2009, 2010). The construct containing an intact *Drd1a* promoter followed by the A-CREB sequence was injected into C57BL/6N prezygotes, which were then transferred into foster mothers. Resulting offspring were screened by Southern blotting for mice harboring the integrated transgene and “founder” animals with different numbers of integrated transgene copies were identified (Figure 1B). No early lethality, morbidity, or apparent deficits were observed in lines carrying 1, 2, or 4 copies of the transgene. We selected one of the strains with 4 transgene copies for further characterization.

The specificity of transgene expression was validated by immunohistochemistry with antibodies against the FLAG moiety located at the C-terminus of A-CREB (Figure 1C). Stained cells are observed in the basal ganglia including the caudate/putamen and NAc as well as lower layers of the sensorimotor, cingulate and limbic cortex. Importantly, double immunofluorescence staining of dynorphin, a marker of direct pathway medium spiny neurons, and A-CREB shows perfect overlap (Figure 1D). Thus, the expression of A-CREB is specific to prodynorphin-expressing neurons of the direct pathway, which is in agreement with D1R expression in the mature striatum (Gerfen et al., 1990; Noori et al., 2012).

**Figure 2:**
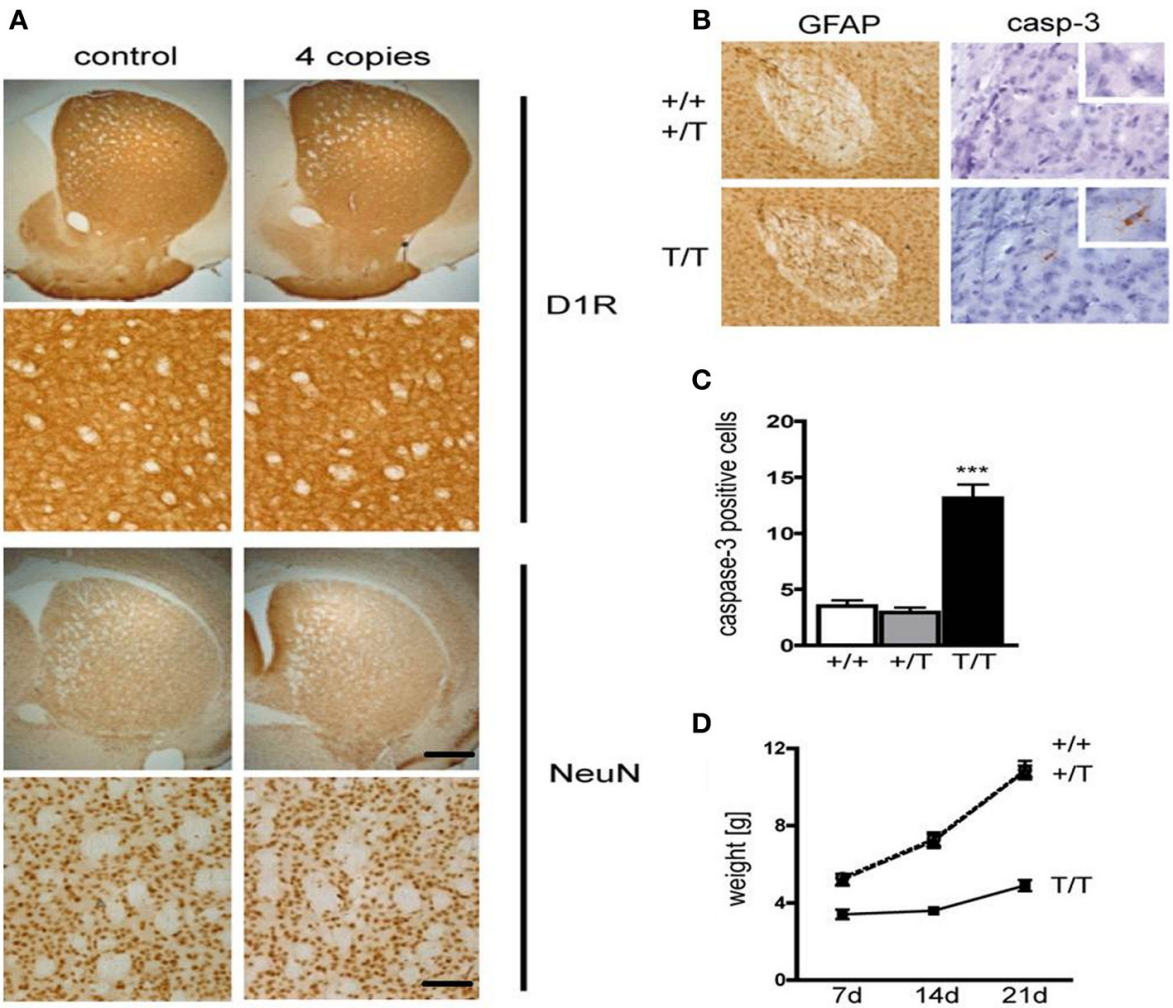
**Expression of D1-A-CREB transgene causes no significant increase in apoptosis or cell loss. (A)** Immunohistochemical staining of coronal sections with antibodies against the dopamine receptor D1 (D1R) and the neuronal marker NeuN revealed no loss of cells or decrease in D1R abundance. **(B)** In contrast, D1-A-CREB mice carrying two transgenes (T/T) show an increase of GFAP (brown) and cleaved caspase-3 staining in comparison to wild-type (+/+) and single-transgene mice (+/T). **(C)** Quantification of caspase-3 positive cells in striata of coronal section in wild-type, single transgene and double transgene mice. **(D)** Homozygous mice show reduced weight gain in comparison to wild-type and single transgene. Data are presented as mean + s.e.m., *p*-value of *t*-test (^***^P < 0.001). Scale bars **(A)** 50 μm; **(B)** 70 μm (left panel), 30 μm (right panel), 15 μm (insert).

It has been previously demonstrated that CREB is essential for survival of neurons (Mantamadiotis et al., 2002; Jancic et al., 2009). Staining of coronal sections containing the striatum from D1-A-CREB (with 4 copies) or wild-type mice with antibodies against the D1R or the neuronal marker NeuN revealed no neurodegeneration or decrease in D1R abundance (Figure 2A). Cross-breeding of two different strains carrying 4 copies of the transgene each resulted in transgenic mice with higher A-CREB expression with a total of 8 A-CREB encoding sequences), which showed mild neuroinflammation. Immunohistochemical staining detected an increase in GFAP, a marker of activated astroglia (Figure 2B), as well as increased number of cells with active caspase 3, an apotosis marker (Figure 2C) in mice carrying two A-CREB transgenes, which was accompanied by attenuated weight gain in the first 4 weeks after birth. In mice carrying a single transgeneno neuroinflammation or increased cell death were observed (Figure 2D). In summary, we generated a novel transgenic mouse line with a selective expression of A-CREB in D1R-containing neurons without any obvious behavioral deficits or neurodegeneration.

### Basal expression profiling and phenotype of D1-A-CREB mice

**Figure 3:**
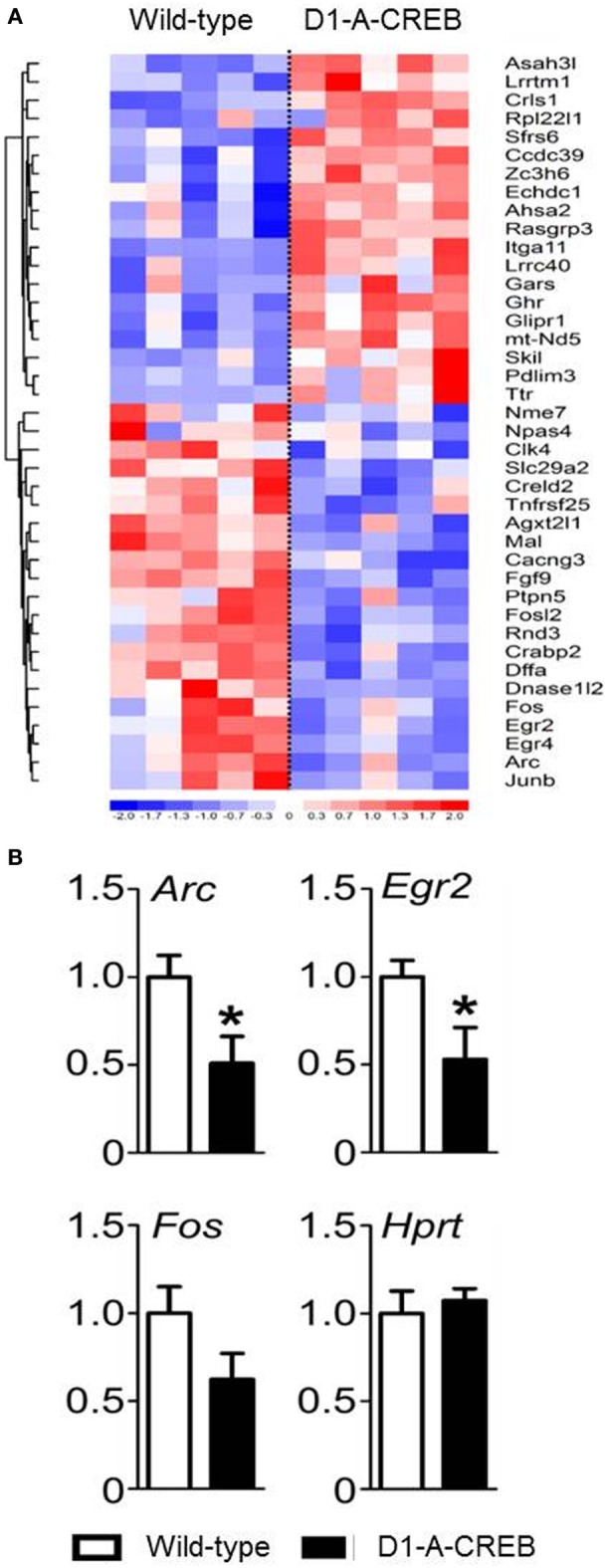
**Effect of A-CREB transgene on the induction of activity-dependent genes. (A)** The heatmap summarizes results from gene expression profiling in the striatum of wild-type (*n* = 5) and D1-A-CREB (*n* = 5) mice using Illumina MouseWG-6 v2 BeadChip arrays. The 40 transcripts included in the heatmap are the top 20 most significantly increased and 20 most decreased in abundance according to the signal-to-noise metric from GSEA 2.0. Each column represents one array and one animal. The color corresponds to a ratio of fold-change to standard error according to the scale shown below the heatmap. The results were clustered (neighbor-joining), Euclidean shortest distance correlations between transcript profiles are represented by the dendrogram on the left. **(B)** Validation by quantitative PCR (qPCR). The bars represent transcript abundance normalized to the levels observed in control animals. Expression of *Arc*, *Egr2*, and *Fosb* in naïve conditions in wild-type and D1-A-CREB mice. Abundance of the “house-keeping” *Hprt* transcript was identical in wild-type and D1-A-CREB mice. Data are presented as mean + s.e.m., *P*-value of *t*-test (^*^P < 0.05).

We performed gene expression profiling on the striatum from naïve D1-A-CREB animals and wild-type controls on Illumina MouseWG-6 v2 BeadChip arrays. Normalized expression values were analyzed using GSEA 2.0 using the signal-to-noise metric. The 40 transcripts with most significant difference in abundance between D1-A-CREB and wild-type animals (20 increased and 20 decreased) are shown in Figure 3A. Overall, the differences in striatal gene expression profiles between wild-type and D1-A-CREB mice were moderate, and the largest ones shown in Figure 3A are <2.5 fold. Several activity-dependent genes, like *Egr2* or *Arc* had lower abundance in naïve D1-A-CREB animals compared to controls (see bottom of the heatmap in Figure 3A), and this finding was validated by qPCR (Figure 3B). There were no changes in the expression of *Pdyn*, *Penk* or genes encoding the DA receptors and no significant increase in the *Crem* transcript abundance.

**Figure 4:**
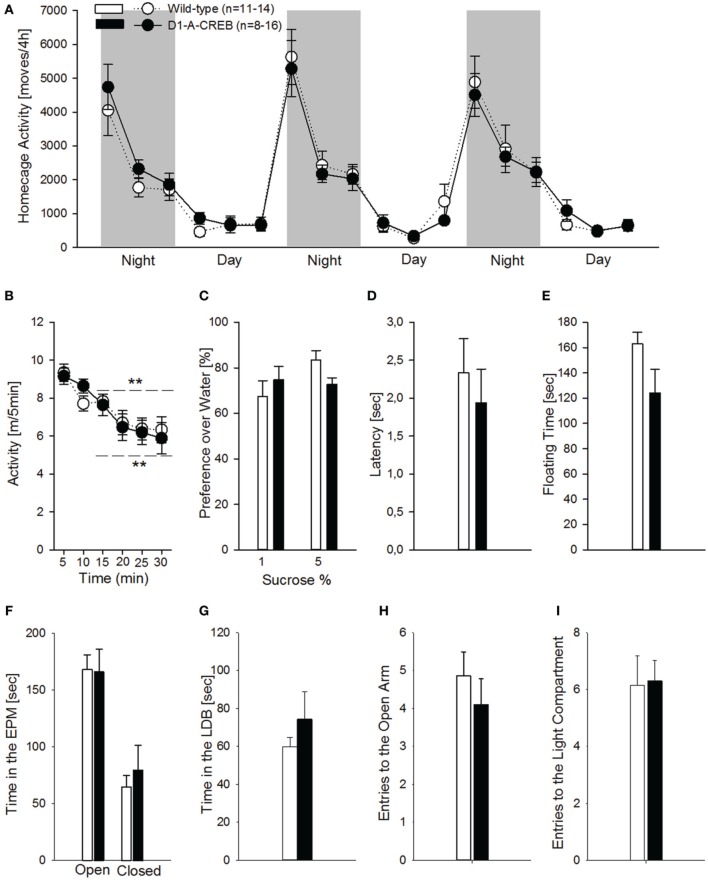
**Locomotor, anxiety- and depressive-like responses in D1R-A-CREB mice. (A)** Spontaneous home cage locomotor activity measured by the e-motion system is indistinguishable between wild-type (*n* = 11) and D1-A-CREB mice (*n* = 8) *genotype* effect [*F*_(1, 102)_ = 0, *p* = 1]. Two-Way ANOVA indicates a *phase* effect [*F*_(1, 102)_ = 141.8, *P* < 0.0001] and all day points are significantly different from all night points *Phase* × *time point* interaction effect [*F*_(2, 102)_ = 18.5, *P* < 0.0001]. **(B)** Habituation to novelty in activity chambers in wild-type (*n* = 14) and D1-A-CREB (*n* = 13) mice. During the first 30 min exposure both genotypes displayed a progressive decrease in locomotor activation indicating habituation to novelty. *Habituation* effect [*F*_(1, 125)_ = 21.2, *P* < 0.001] **(C)** Preference for sucrose in wild-type (*n* = 6–7) and D1-A-CREB (*n* = 6–8) mice. Both genotypes displayed similar preferences for 1 or 5% sucrose solutions. ANOVA analysis did not indicate any significance for *Genotype* [*F*_(1, 23)_ = 0.1, *P* = 0.7], *sucrose solutions* [*F*_(1, 23)_ = 2.7, *P* = 0.1] or *Genotype* × *solutions* interaction [*F*_(1,23)_ = 3.8, *P* = 0.1] effects. **(D,E)** During the second exposure to the FST, both the latency to the first episode of immobility [*t*_(26)_ = 0.6, *P* = 0.5] and the immobility time during the last 4 min test session [*t*_(26)_ = 1.7, *P* = 0.1] was similar in all mice. **(F)** Anxiety-related behavior is not different between both genotypes during the Elevated plus-maze test. The time spent in the open arms of the maze is almost identical in both genotypes (wild-type *n* = 14, D1-A-CREB *n* = 11), and Two-Way ANOVA indicated no *genotype* [*F*_(1, 157)_ = 0.09, *P* = 0.7 but an *arm* effect: *F*_(1, 157)_ = 3.5, *P* < 0.001]. **(G)** Similarly, in the light-dark box test, the time spent in the light area is not different between wild-type (*n* = 13) and D1-A-CREB (*n* = 13) mice [*t*_(26)_ = −1.5; *P* = 0.1]. **(H,I)** The number of entries into the open arm of the elevated plus maze and the lit part of the light dark box did not differ between genotypes [**E**: *t*_(23)_ = 0.8; *P* = 0.4 and **F**: *t*_(24)_ = −0.1; *P* = 0.9]. All data represent mean ± s.e.m. (^**^) indicates *P* < 0.05 and 0.01 vs. 5 m.

Studies involving CREB manipulations in rodent models have demonstrated alterations in motor control, anxiety and depression-like responses. To test the possible occurrence of such alterations in the *D1-A-CREB* transgenic mice, we assessed motor, anxiety-like and depression-like behavior. Hence we measured spontaneous home cage activity, habituation to novelty, elevated plus maze and, light-dark box behavior, as well as sucrose preference and behavior in the FST. In the home cage, both genotypes displayed identical typical diurnal pattern of activity, characterized by increased activity during the dark phase compared to the resting, light phase of the day (Figure 4A). When tested in an unfamiliar environment (the above described activity chamber), again no differences could be observed in terms of exploratory behavior, and both genotypes displayed similar horizontal activity and habituation (Figure 4B). Depressive-like responses were assessed by the sucrose preference test and FST (Figures 4C–E). Mice were exposed to a 1 or 5% sucrose solution for 15 min and the preference over water was measured. Both genotypes displayed high preferences over water for both sucrose solutions tested (Figure 4C). During the FST, we did not find genotype differences in the latency to the first floating episode (Figure 4D) or the total time spent floating across the session (Figure 4E). To assess whether the D1-A-CREB mice were more responsive to anxiogenic environments, we used the elevated plus maze and the light-dark box tests. D1-A-CREB mice exhibited similar responses compared to wild-type mice in both tests as reflected in the time spent in the exposed versus non-exposed arms of the elevated plus maze (Figure 4F), and in the light compartment of the light-dark box (Figure 4G), as well as in the entries made into the open arm (Figure 4H) or light compartment (Figure 4I).

### Cocaine-induced behavioral responses and gene expression

**Figure 5:**
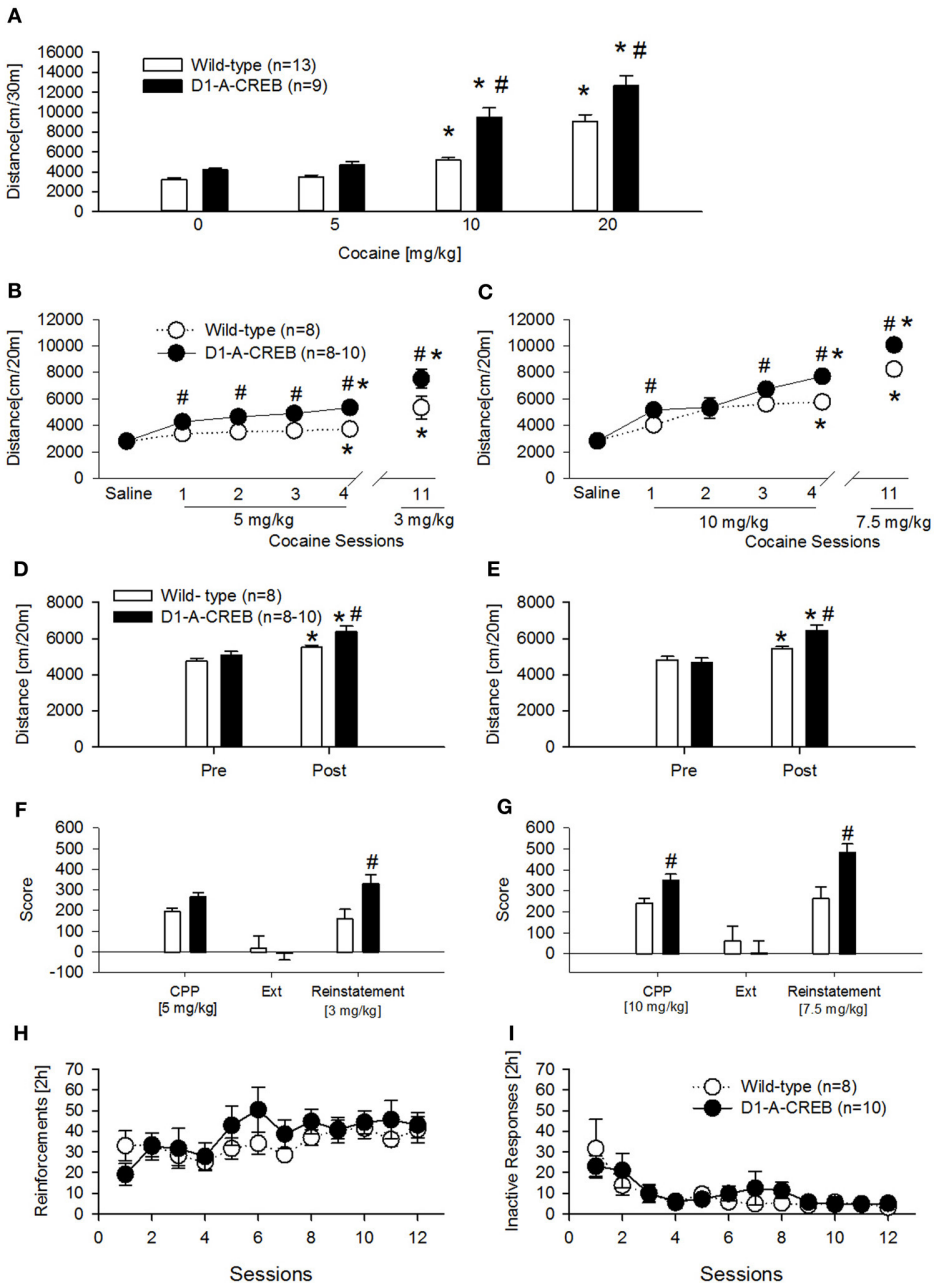
**Cocaine-induced behavioral effects in D1-A-CREB mice. (A)** Cocaine induced a higher increase in locomotor activity in the activity box at doses of 10 and 20 mg/kg cocaine in D1-A-CREB (*n* = 9) mice compared with wild-type (*n* = 13) mice. Two-Way ANOVA indicated a *Genotype* effect *F*_(1, 19)_ = 27.5, *P* < 0.001, a *Dose* effect *F*_(3, 57)_ = 132.3, *P* < 0.001, and a *Genotype* × *Dose* interaction *F*_(3, 57)_ = 4.3, *P* < 0.01. **(B,C)** Cocaine-induced development and expression of behavioral sensitization. At the dose of 5 mg/kg **(B)**, wild-type and D1-A-CREB mice showed development of cocaine sensitization [*Sensitization* effect *F*_(5, 70)_ = 29.9, *P* < 0.001], but D1-A-CREB mice expressed a significantly higher response to repeated cocaine injections and after a drug free interval (day 11) than the wild-type mice. Two-Way ANOVA indicated *genotype* [*F*_(1, 14)_ = 21.9, *P* < 0.0005] and *Genotype* × *Sensitization* [*F*_(3, 70)_ = 2.3, *P* < 0.05] effect. Similarly, at the dose of 10 mg/kg **(C)** both development and the expression of behavioral sensitization were stronger in D1-A-CREB than in wild-type mice. Two-Way ANOVA indicated *Genotype* [*F*_(1, 16)_ = 10.3, *P* < 0.005], *Sensitization* [*F*_(5, 80)_ = 69.7, *P* < 0.0001] and *Genotype* × *Sensitization* [*F*_(5, 80)_ = 2.8, *P* < 0.05] effects. **(D,E)** All mice exhibited a conditioned locomotion in the CPP boxes after the cocaine treatment [Two-Way ANOVA, *Conditioning* effect *F*_(1, 14)_ = 29.6; *P* < 0.001 and *F*_(1, 14)_ = 75.2; *P* < 0.001 for **(D,E)** respectively]. This conditioned response was higher in D1-A-CREB mutants compared with wild-type mice [Two-Way ANOVA indicated a *Genotype* effect *F*_(1, 14)_ = 9.9; *P* < 0.01 for **(D)** and a *Genotype* × *Conditioning* effect: *F*_(1, 14)_ = 21.9; *P* < 0.0005 for **(E)**]. **(F,G)** Cocaine-induced CPP, extinction and reinstatement. Although both genotypes exhibited a significant cocaine-induced CPP, the D1-A-CREB mice displayed an increased—though non-significant- preference for the cocaine-paired compartment compared to wild-type mice at the dose of 5 mg/kg, which became significant when the mutants were conditioned with the dose of 10 mg/kg [*F*_(1, 16)_ = 3.3, *P* < 0.05]. There were no genotype differences during the extinction test, as indicated by the similar reduction of the time spent in the cocaine-paired floor after extinction training. However, after extinction, a challenge injection of cocaine [3 and 7.5 mg/kg, i.p. in **(D,E)**, respectively] induced an increased reinstatement of the CPP in the D1-A-CREB mice, as indicated by the significantly stronger CPP score *Genotype* × *Score* [*F*_(2, 32)_ = 6.4, *P* < 0.005] interaction effect. **(H,I)** Cocaine self-administration. **(H)** Both genotypes learned to self-administer cocaine as indicated by the number of reinforcements across 12 consecutive sessions with a 0.5 mg/kg per infusion training dose (wild-type *n* = 8 and D1-A-CREB *n* = 10 mice). Two-Way ANOVA indicated a *time* [*F*_(11, 176)_ = 2.809; *P* < 0.01] but not a *Genotype* [*F*_(1, 16)_ = 0.5, *P* > 0.05] or *Genotype* × *time* interaction [*F*_(11, 176)_ = 1.1, *P* > 0.05] effects. **(I)** The inactive lever pressing was not different between genotypes. Two-Way ANOVA indicated a *Time* effect [*F*_(11, 176)_ = 6, *P* < 0.01], but not a *Genotype* [*F*_(1, 16)_ = 1.1, *P* > 0.05] or *Genotype* × *time* interaction [*F*_(11, 176)_ = 0.7, *P* > 0.05] effects. Data represent mean ± s.e.m. ^*^P < 0.05 compared with 0. ^#^*P* < 0.05 compared with wild-type mice.

First we assessed the dose-response effects to acute cocaine injections (Figure 5A). Following saline or 5 mg/kg cocaine injections, the transgenic group did not differ from the wild-type mice but D1-A-CREB mice showed increased locomotor responses to cocaine at the doses of 10 and 20 mg/kg when compared to wild-type mice.

A second and third cohort of mice was injected daily with cocaine (two doses—5 and 10 mg/kg, i.p.) for 4 alternating days to test the development of CPP and behavioral sensitization simultaneously (Figures 5B,C). Both cocaine doses induced a higher increase in locomotor activity in D1-A-CREB mice compared to wild-type groups (Figures 5B,C, cocaine sessions 1–4). After a drug free interval of 11 days, all mice further increased their sensitized response to cocaine, and again a genotype effect was observed as D1-A-CREB mice exhibited a more robust response than wild-type mice at both doses tested. We also measured conditioned locomotion, the increase in locomotor response during exposure to the cocaine-paired context relative to the same context prior to cocaine administration. All mice showed an increased locomotion after the cocaine administration compared to the baseline locomotion before the beginning of cocaine treatment, indicating cocaine-conditioned locomotion (Figures 5D,E). However, this response was more robust in D1-A-CREB mice, indicating higher conditioned locomotion (Figures 5D,E, “post”). These results indicate augmented psychomotor and conditioned responses to repeated intermittent injections of cocaine in D1-A-CREB mice and suggest that this enhanced drug sensitivity may be also influencing the rewarding effects of cocaine.

Indeed the transgenic mice displayed an increased—though non-significant—preference for the cocaine-paired compartment compared to wild-type mice at the dose of 5 mg/kg (Figure 5F, CPP). The CPP response became significant when the mutants were conditioned with the dose of 10 mg/kg (Figure 5G, CPP). In order to assess the role of CREB in the persistence of cocaine-seeking behavior we next studied the extinction of both CPP responses by saline injections in the previously drug-paired environment. All mice showed no CPP anymore after 8 extinction sessions (Figures 5F,G, Ext). We then studied cocaine-seeking behavior by re-exposure to the drug after the extinction period. A priming dose of cocaine (3 and 7.5 mg/kg) reinstated CPP in all groups but this effect was more robust in D1-A-CREB mice compared to wild-types regardless of the cocaine dose tested (Figures 5F,G, Reinstatement). These results indicate that CREB signaling in D1R expressing neurons influences the conditioned rewarding properties of cocaine.

To further examine the rewarding aspects of cocaine, another cohort of mice was studied for cocaine's primary reinforcing properties under operant self-administration conditions. Contrary to what was observed in the CPP experiment, when the mice were trained to self-administer cocaine (0.5 mg/kg per infusion) for 12 consecutive sessions, the acquisition and maintenance of stable reinforcement earning was indistinguishable between both genotypes (Figure 5H). Responses on the inactive lever were almost identical in all mice (Figure 5I).

**Figure 6:**
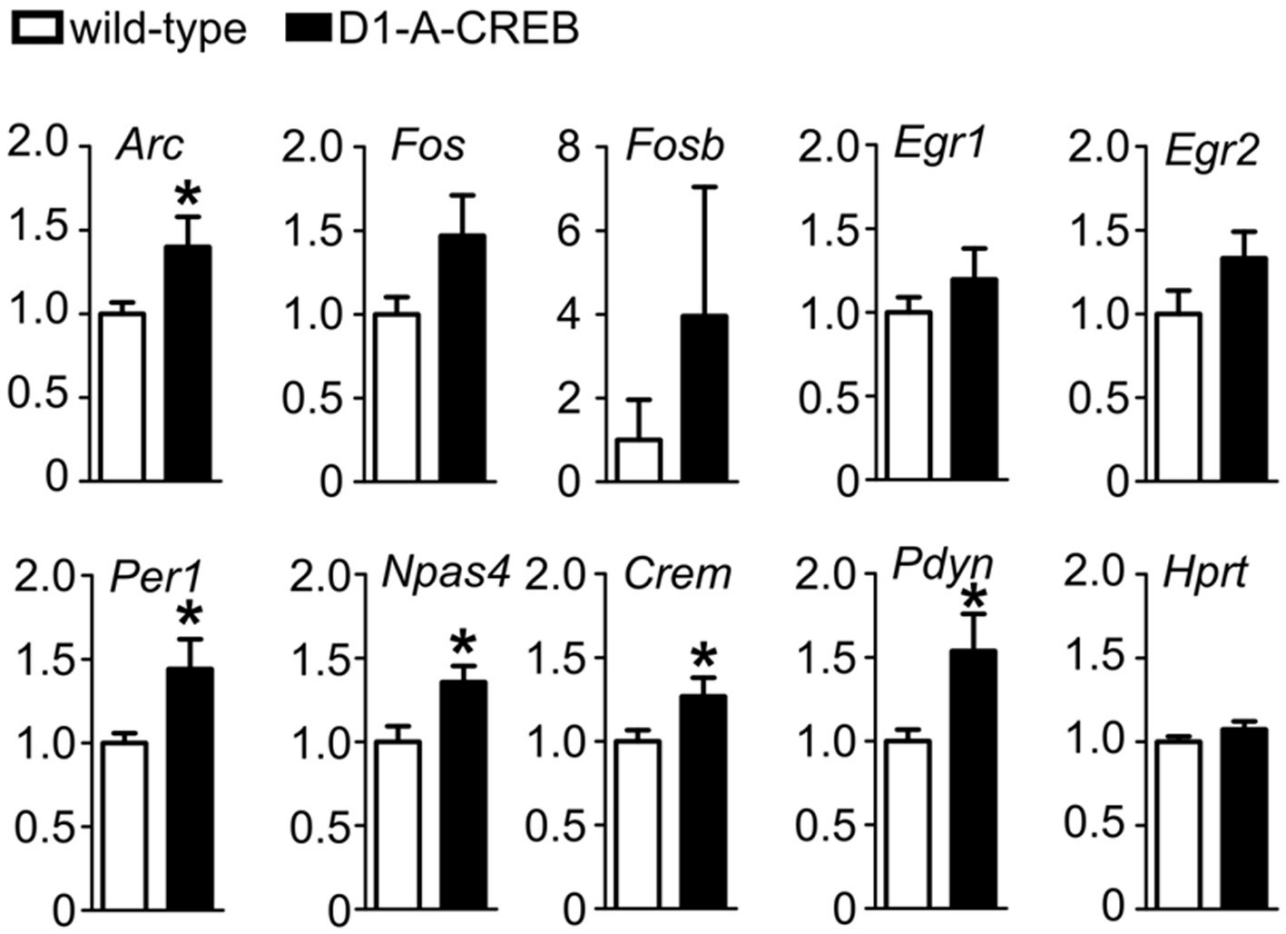
**Effect of A-CREB transgene on cocaine-dependent induction of activity-dependent genes**. The graphs show mean abundances (8 mutants and 13 controls) of activity-dependent transcripts in mice 1 h after 7.5 mg/kg i.p. cocaine injection at the start of the reinstatement test. Values are normalized to control levels. Significant difference *P* < 0.05 (*t*-test) between wild-type and D1-A-CREB mice is labeled with a “^*^.”

Finally, we tested the effects of A-CREB on regulation of gene expression after cocaine treatment. We measured transcript levels in the striatum of D1-A-CREB and wild-type mice after the reinstatement of CPP 1 h after injection of 7.5 mg/kg cocaine (Figure 6). We found that mRNA levels of activity-dependent transcripts *Arc, Npas4, Per1* as well as *Crem* and *Pdyn* were higher in transgenic mice than wild-type controls. The mean levels of *Fos, Fosb*, *Egr1*, and *Egr2* were also increased in D1-A-CREB mice compared to wild-type animals, but these differences were not statistically significant.

## Discussion

We used several behavioral paradigms to model different aspects that are of relevance to cocaine addiction, and found that expression of A-CREB in D1R expressing neurons enhances the acute psychomotor properties of cocaine and augments cocaine conditioned responses such as development and expression of behavioral sensitization, conditioned locomotion, CPP and priming-induced reinstatement of an extinguished CPP response, while the primary reinforcing effects of cocaine, as assessed in the self-administration paradigm was not affected at a training dose of 0.5 mg/kg/infusion. However, A-CREB expression did not cause attenuated activity-dependent transcription when cocaine was given to induce the reinstatement of an extinguished CPP response suggesting that A-CREB caused an adaptation in the cell signaling processes rather than a blockade of immediate-early gene expression. In conclusion, we identify the activity of CREB in D1R expressing neurons as responsible for its previously described role in psychostimulants-induced reward processes (Carlezon et al., 1998; Walters and Blendy, 2001; Barrot et al., 2002; Larson et al., 2011; Madsen et al., 2012).

Using a BAC-derived transgene we achieved expression of the A-CREB protein exclusively in dynorphin-containing cells in the striatum which is in agreement with the pattern of D1R expression in the mature striatum (Gerfen et al., 1990; Noori et al., 2012). The transgene contained the same promoter as we had recently described and replicated the previously observed specificity (Novak et al., 2010). This confirms the reliability of BAC-derived transgenes (for further discussion see Nelson et al., 2012), and their relative independence of the site of integration effects. Unlike the complete CREB and CREM deletion, A-CREB expression was not associated with observable neuron loss or gliosis. The increase in number of apoptotic cells observed in mice carrying two D1-A-CREB transgenes was relatively minor compared to progressive degeneration of the entire dorsal striatum found in double CREB/CREM KO mice (Mantamadiotis et al., 2002; Lemberger et al., 2008) or expression of dominant-negative CREB in the hippocampus (Jancic et al., 2009). It should be noted that even a fraction of CREB/CREM activity (i.e., presence of one *Crem* allele) was shown to be sufficient to prevent neuronal death. Additionally, in case of previous mouse models the mutation affected the majority of striatal neurons, rather than specifically D1-expressing cells.

The D1-A-CREB mice had no apparent developmental impairments and no obvious phenotype in spontaneous motor control or anxiety- and depressive-like behaviors. Other mutants models like the *C*reb1^*DARPP32Cre*^ or *Creb1^EMX1Cre^* also exhibit normal anxiety-like behaviors (McPherson et al., 2010; Madsen et al., 2012) but in the *Creb1^NesCre^ mice* (Valverde et al., 2004) and the α/Δ CREB strain (Pandey et al., 2004; Valverde et al., 2004) enhanced anxiety responses had been reported. The motor phenotype also varies accordingly to the genetic model tested. Thus, hypolocomotion was observed in *Creb1^EMX1Cre^* and *Creb1^NesCre^* strains, even though spontaneous locomotor activity was normal in the latter case (Valverde et al., 2004; McPherson et al., 2010) and mice hypomorphic for *CREB1* had reduced spontaneous locomotor activity (Valverde et al., 2004). In addition to the studies involving CREB-deficient mouse models, others approaches have indicated that reduction in accumbal CREB activity is associated with reduced depression-like behavior in rats as assessed by the sucrose preference test and FST (Pliakas et al., 2001; Green et al., 2010). In our mouse model, responses in these tests were not found to be altered.

The fact that the D1-A-CREB mice do not display such altered phenotypes suggest that expression of CREB within D1R neuronal population does not mediate these responses and does also rule out potentially confounding influences in subsequent measures on cocaine-induced behaviors. Although transgenic mice displayed no obvious phenotype in locomotor activity, anxiety- and depressive-like behavior they showed a lower abundance of *Egr2* and *Arc* striatal transcripts compared to control mice suggesting that the activity of these immediate early genes is also not critical for those behaviors. In respect to *Egr2* this suggestion is supported by findings in *Egr2*-deficient mice that display no signs of locomotor, exploratory or anxiety disturbances (Poirier et al., 2007).

The D1-A-CREB mice exhibited increased locomotor responses to cocaine treatment. Furthermore, they showed stronger psychomotor sensitization than wild-type littermates. A similar phenotype has been observed in rats injected intrastriatally with a virus vector expressing a dominant negative CREB variant (Brown et al., 2011) or with partial expression of a dominant negative CREB in the dorsal striatum (Fasano et al., 2009). In contrast, studies with genetic inactivation of *Creb1* showed no alteration (Kreibich and Blendy, 2004; Valverde et al., 2004; Bilbao et al., 2008; McPherson et al., 2010) or increased (Walters and Blendy, 2001; Madsen et al., 2012) sensitivity. These data indicate that within striatal areas, the selective expression of an A-CREB in D1R expressing neurons is sufficient to alter the psychomotor effects of cocaine. Additionally, the D1-A-CREB mice also displayed increased cocaine-induced conditioned responses. Thus, the conditioned locomotion, preference and seeking response induced by repeated pairings of cocaine injections was augmented in the transgenic animals. Previous studies that investigated the role of CREB in CPP showed in rats that mutant CREB expressed in the NAc shell and mice with hypomorphic CREB1, respectively, exhibit an increased CPP response (Carlezon et al., 1998; Walters and Blendy, 2001) but no cocaine-induced reinstatement (Kreibich and Blendy, 2004). The phenotype of D1-A-CREB mice is similar to that of the *Camk4^D1Cre^* animals where the CaMKIV kinase, a principal CREB activator, is ablated in D1R expressing neurons. The selective loss of CaMKIV led to increased levels of cocaine sensitization, enhanced cocaine-induced conditioned place preference and an augmented reinstatement response (Bilbao et al., 2008). Similarities between D1-A-CREB and *Camk4^D1Cre^* are also apparent on the gene expression. Thus, expression of A-CREB was associated with increased abundances of several activity-regulated transcripts (e.g., Arc, Per1 or Npas4) in the striatum of mice after reinstatement of cocaine-induced CPP. Additionally we observed an increase in abundance of *Pdyn* transcript, which could be indicative of sustained increase in activity. *Pdyn* expression changes are delayed and more persistent compared to activity-regulated transcripts (Piechota et al., 2012). Virally mediated downregulation with mCREB has been associated with reductions, rather than elevations, in *Pdyn* (Carlezon et al., 1998). However, in our study, the up-regulation in *Pdyn* levels was observed after the priming-induced reinstatement of cocaine-seeking behavior in the CPP paradigm. Therefore, we assume that the alterations observed are more related to interactions between CREB and cocaine-induced conditioning, rather than a compensatory adaptation in response to chronic CREB down-regulation. The finding of increased cocaine-induced activity-regulated transcripts in the transgenic mice is intriguing. However, we propose that A-CREB caused an adaptation in the cell signaling processes rather than a blockade of immediate-early gene expression. Indeed, the D1R gene promoter becomes active relatively early in development, which may allow substantial time for adaptations to the transgene. In this respect the use of an inducible D1-A-CREB mouse mutant would be of interest-On the other hand, without specifically targeting CREB function in D2R containing neurons it remains uncertain if the alterations in gene expression are due tospecific A-CREB expression in the D1R neurons or just due to an approximate 50% inactivation oftotal CREB in the striatum. Nevertheless, any compensatory gene expression changes induced in D2 and other types of neurons in the mutants that could potentially account for the observed alterations in cocaine-induced activity-regulated transcripts is unlikely, since psychostimulants-induced changes in gene regulation in the striatum occur preferentially in D1Rs from the direct pathway striatonigral neurons (Lobo and Nestler, 2011).

In our transgenic model expressing A-CREB in D1R expressing neurons the primary reinforcing effects of cocaine were intact for the training dose of 0.5 mg/kg/infusion as revealed by the operant self-administration experiment. This is in line with a previous report (Larson et al., 2011) which showed no alteration in self-administration following CREB down-regulation in the NAc. However, in this study, although NAc shell mCREB did not alter self-administration on a fixed ratio schedule of reinforcement over a broad range of cocaine doses, direct down-regulation of CREB using a CREB-RNAi did reduce low dose self-administration, and additionally reduced the motivation for cocaine in a progressive ratio (PR) test. In contrast, CREB over-expression enhanced cocaine self-administration, facilitated the motivation for cocaine (PR), and increased cocaine-induced reinstatement. Another study (Hollander et al., 2010) has also shown that micro-RNA mediated facilitation of CREB in the dorsal striatum reduces cocaine self-administration. Therefore, it is also possible that the lack of effect on self-administration in our study was due to competing effects of CREB down-regulation in both dorsal and ventral striatal regions.

In conclusion, by the use of a novel transgenic mouse model and several paradigms modeling drug-induced phenotypes that are of relevance for addictive behavior, we extend previous findings by showing a specific role of CREB in D1R expressing neurons in regulating behavioral responses to cocaine. In particular, inhibition of CREB in D1R expressing neurons facilitates the acute psycho stimulant effects of cocaine, the expression of behavioral sensitization, conditioned responses to cocaine and priming-induced reinstatement of CPP. Our study highlights the importance of identifying a selective involvement of CREB in a given signaling pathway (D1R neurons) which is particularly required for cocaine-induced sensitivity and conditioned responses.

## Author contributions

Ainhoa Bilbao, Claus Rieker, Nazzareno Cannella, David Engblom, Günther Schütz, Rainer Spanagel, and Jan R. Parkitna designed research; Ainhoa Bilbao, Claus Rieker, Nazzareno Cannella, David Engblom, Ryszard Przewlocki, Slawomir Golda, Marcin Piechota, Michal Korostynski, and Jan R. Parkitna performed research; Ainhoa Bilbao, Claus Rieker, Nazzareno Cannella, Slawomir Golda, Marcin Piechota, Michal Korostynski, Rainer Spanagel, Rosanna Parlato, and Jan R. Parkitna analyzed data; Ainhoa Bilbao, Rainer Spanagel, and Jan R. Parkitna wrote the paper.

### Conflict of interest statement

The authors declare that the research was conducted in the absence of any commercial or financial relationships that could be construed as a potential conflict of interest.
