# Corrigendum: CREB activity in dopamine D1 receptor expressing neurons regulates cocaine-induced behavioral effects

**DOI:** 10.3389/fnbeh.2014.00239

**Published:** 2014-07-09

**Authors:** Ainhoa Bilbao, Claus Rieker, Nazzareno Cannella, Rosanna Parlato, Slawomir Golda, Marcin Piechota, Michal Korostynski, David Engblom, Ryszard Przewlocki, Günther Schütz, Rainer Spanagel, Jan Rodriguez Parkitna

**Affiliations:** ^1^Institute of Psychopharmacology, Central Institute of Mental Health, Faculty of Medicine Mannheim, University of HeidelbergHeidelberg, Germany; ^2^Department of Molecular Biology of the Cell I, DKFZ-ZMBH Alliance, German Cancer Research CenterHeidelberg, Germany; ^3^Institute of Applied Physiology, University of UlmUlm, Germany; ^4^Department of Medical Biology, Institute of Anatomy and Cell Biology, University of HeidelbergHeidelberg, Germany; ^5^Department of Molecular Neuropharmacology, Institute of Pharmacology of the Polish Academy of SciencesKrakow, Poland

**Keywords:** CREB, dominant negative CREB, dopamine receptor D1, activity-dependent gene expression, cocaine-related behavior, addiction

## Conflict of interest statement

The authors declare that the research was conducted in the absence of any commercial or financial relationships that could be construed as a potential conflict of interest.

